# Traditional Chinese Medicine Tanreqing Targets Both Cell Division and Virulence in *Staphylococcus aureus*

**DOI:** 10.3389/fcimb.2022.884045

**Published:** 2022-04-27

**Authors:** Weifeng Yang, Kaiyu Cui, Qian Tong, Shuhua Ma, Yanan Sun, Gaiying He, Dongying Li, Longfei Lin, Biljana Blazekovic, Sylvie Chevalier, Yuanhong Wang, Qing Wei, Yi Wang

**Affiliations:** ^1^Experimental Research Center, China Academy of Chinese Medical Sciences, Beijing, China; ^2^School of Biological Engineering and Food Science, Hubei University of Technology, Wuhan, China; ^3^Institute of Chinese Materia Medica, China Academy of Chinese Medical Sciences, Beijing, China; ^4^Department of Pharmacognosy, Faculty of Pharmacy and Biochemistry, University of Zagreb, Zagreb, Croatia; ^5^Laboratory of Microbiology Signals and Microenvironment, Normandy University, University of Rouen Normandy, Evreux, France; ^6^College of Horticulture and Landscape, Tianjin Agricultural University, Tianjin, China; ^7^Nanchang Institute of Technology, Nanchang, China

**Keywords:** Tanreqing, cell division, virulence, inhibition, *Staphylococcus aureus*

## Abstract

*Staphylococcus aureus* has been recognized as an important human pathogen and poses a serious health threat worldwide. With the advent of antibiotic resistance, such as the increased number of methicillin-resistant *Staphylococcus aureus* (MRSA), there is an urgent need to develop new therapeutical agents. In this study, Chinese traditional medicine Tanreqing (TRQ) has been used as an alternative treating agent against MRSA and we aim to unravel the mode of action of TRQ underlying MRSA inhibition. TRQ treatment affected numerous gene expression as revealed by RNA-seq analysis. Meanwhile, TRQ targeted cell division to inhibit cell growth as shown by illumination microscopy. Besides, we confirmed that TRQ downregulates the expression of virulence factors such as hemolysin and autolysin. Finally, we used a murine model to demonstrate that TRQ efficiently reduces bacterial virulence. Altogether, we have proved TRQ formula to be an effective agent against *S. aureus* infections.

## Importance

*Staphylococcus aureus* is an important human pathogen that poses a serious health threat worldwide. To achieve a successful colonization, this bacterium produces a large number of virulence determinants that interfere with the host immune system. At the same time, the advent of multiple antibiotic resistance of *S. aureus* has urged the development of novel antimicrobial agents. We aimed to use Chinese traditional medicine Tanreqing (TRQ) as an alternative antimicrobial agent against *S. aureus*. Using RNA-seq analysis in combination with super-resolution microscopy, we found that TRQ not only affects expression of virulence genes, but also targets cell division to inhibit cell growth, which finally leads to cell death. We demonstrated *in vivo* that TRQ efficiently reduces bacterial virulence in a murine model. Altogether, we have proved TRQ to be an effective and specific agent to combat *S. aureus* infections.

## Introduction

*Staphylococcus aureus* is one of the important community-based human pathogens that causes worldwide life-threating infections (Lowy, 1998; Knox et al., 2015). The basis for this is multifactorial and attributed to the emergence of multidrug resistance, enhanced virulence and versatile adaptability (Deleo and Chambers, 2009). Since the 1980s, the increased number of methicillin-resistant *Staphylococcus aureus* (MRSA) infections has caused a serious public health threat (Fridkin et al., 2005; Knox et al., 2015). In view of these facts, there is urgent need for the development and search for novel agents that target this pathogen (Arias and Murray, 2009).

Successful *S. aureus* infection depends on the production of a large number of virulence determinants that interfere with the host immune system (Bronner et al., 2004). For instance, *S. aureus* produces several other virulence factors such as hemolysins, leukocidins, proteases, enterotoxins, and immune-modulatory factors (Kuroda et al., 2001; Foster, 2004; Foster, 2005). To initiate infections, *S. aureus* has acquired coordinated expression of virulence genes through signal transduction, mainly *via* two-component systems (TCS), such as the Agr, SaeRS, SrrAB, WalKR and LytRS systems (Bronner et al., 2004; Delaune et al., 2012). These systems respond to environmental clues and consist of a sensor kinase and a response regulator (Bronner et al., 2004). In addition, multiple global regulators, such as SarA, MgrA, and SarZ, have also been characterized to allow *S. aureus* to efficiently adapt to environmental niches and specifically develop infections (Bronner et al., 2004; Chen et al., 2006; Chen et al., 2009).

To survive and proliferate, *S. aureus* has developed exquisite mechanisms for cell division. This process is coordinated by a protein complex called the divisome, the assembly of which is mediated by the conserved tubulin homologue FtsZ (Haeusser and Margolin, 2016). FtsZ is a GTPase and undergoes a GTP-dependent polymerization into filaments to form a ring-like structure known as Z-ring that initiates the separation process (Erickson et al., 2010; Haeusser and Margolin, 2016; Yang et al., 2017). The polymerized FtsZ recruits other cell wall division proteins, such as FtsA, ZipA (Haeusser and Margolin, 2016), FtsK (Yu et al., 1998), FtsL (Robichon et al., 2011; Park et al., 2020), FtsW (Wang et al., 1998), and MurJ (Monteiro et al., 2018), either by direct or secondary physical interactions. All of these cell division proteins are localized at mid-cell and constrict the cell to partition into two (Xiao and Goley, 2016).

In recent years, both virulence and cell division have received extensive studies to develop strategies against *S. aureus* infections (Lampson and Kapoor, 2006; Lock and Harry, 2008; Anderson et al., 2012; Gordon et al., 2013). Such approaches would attenuate infection *via* non-bactericidal pathways and exert less selective pressure to form resistance to the developed antibacterial agents (Clatworthy et al., 2007; Gordon et al., 2013). Among these agents, traditional Chinese medicine (TCM) has gained attractive consideration due to the broad spectrum of secondary metabolites and low potential to develop resistance (Koh et al., 2013; Asfour, 2018; Chong et al., 2018). Previously, we have shown that Tanreqing (TRQ) injection could efficiently inhibit quorum sensing systems in *Pseudomonas aeruginosa* (Yang et al., 2020) and suppress the biofilm formation of *S. aureus* in a mechanism different from that of penicillin (Wang et al., 2011). TRQ injection is a classical formulation prepared from five TCMs including *Scutellariae radix* (Huang Qin), *Lonicerae flos* (Jin Yin Hua), *Forsythiae fructus* (Lian Qiao), *Ursi fel* (Xiong Dan) and *Naemorhedi cornu* (Shan Yang Jiao) (Li et al., 2019). According to TCM theory, TRQ has several activities such as antibacterial, antiviral and anti-inflammation and is widely used in China as a treatment for respiratory tract infection, pneumonia and chronic obstructive pulmonary disease (COPD) (Liu et al., 2016).

In this study, we asked whether TRQ could have an effect against planktonic *S. aureus* strains. Using transcriptome analysis, we found that TRQ reduced the expression of genes encoding virulence factors, transcriptional regulators, and cell division proteins in *S. aureus* at sub-minimum inhibitory concentrations (sub-MIC) *in vitro* and *in vivo*. We have further confirmed that TRQ targets cell division and pathogenesis to attenuate *S. aureus* infection *in vitro* and *in vivo*.

## Results

### RNA-Seq Analysis of TRQ in *S. aureus* Uncovered Comprehensive Changes in Transcription

**Figure 1:**
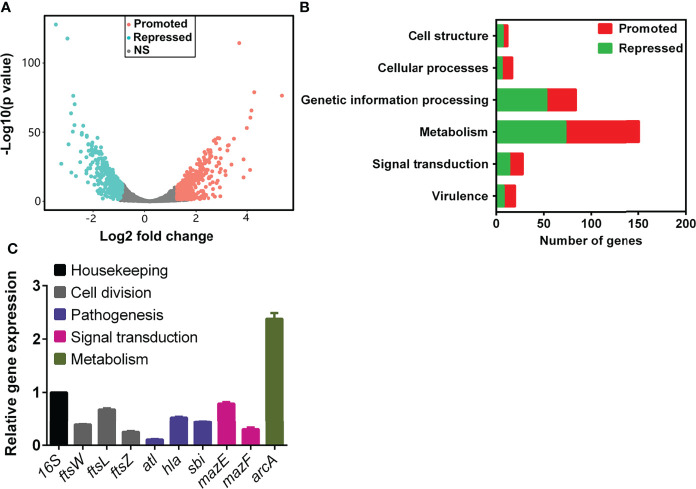
TRQ-treated *S. aureus* regulon analysis. **(A)** Volcano plot of differentially expressed genes in TRQ-treated *S. aureus* cells. NS, not significant. **(B)** Functional classification of differentially expressed genes in TRQ-treated *S. aureus*. Several major classifications were shown including metabolism and virulence, etc. **(C)** Relative gene expression analysis of RNA-seq data using qRT-PCR. Statistical analysis was based on pairwise comparisons (Student’s *t*-test). Error bars represent the mean ± SD.

To gain insight into the effect of TRQ on *S. aureus*, we carried out a global analysis of the transcriptional response upon TRQ treatment under planktonic condition. *S. aureus* cells were harvested at late exponential phase (1.0 of OD_600_) and RNA was extracted and processed according to the recommendations of the Illumina system for RNA-seq analyses. After data qualification control and processing, a comprehensive data set was obtained (**Table S1****)**. TRQ treatment affects the expression of 794 genes (30.5% of the *S. aureus* genome), whereby 418 genes were upregulated and 376 downregulated (*P* value < 0.01) (**Figure 1A**). Functional classification of the affected genes indicates that genes involved in cell structure, metabolism, signal transduction and virulence showed drastic changes at transcriptional level in the TRQ-treated group compared to the untreated control group (**Figure 1B**). To verify the RNA-seq data, qRT-PCR was performed to analyzed nine selected genes and the results demonstrated excellent correlation with the RNA-seq results (**Figure 1C**). Altogether, our data have provided a reliable and reproducible gene expression analysis that would generate insights into the mode of action of TRQ on *S. aureus* planktonic cells.

### TRQ Targeted Cell Division in *S. aureus*

**Figure 2:**
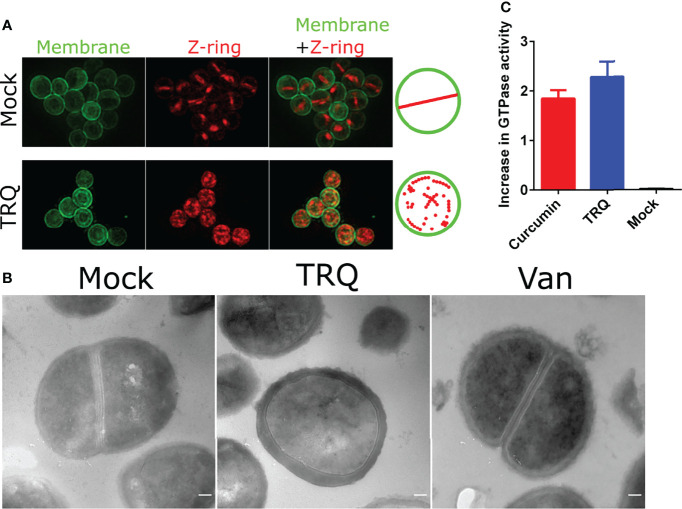
TRQ inhibits cell division in *S. aureus.* ***(*A*)*** Z-ring localization in *S. aureus* upon TRQ treatment (1/16 dilution) compared to mock using structured illumination microscopy (SIM). First column: membranes visualized by Nile Red; second column: Z-ring formation visualized by WGA-488; third column: overlay, membrane and Z-ring visualization. Depictions of Z-ring localization patterns are to the right of the panels **(B)** TEM analysis of TRQ-treated (1/16 dilution) *S. aureus* cells. A negative control (mock) and an antibiotics (Vancomycin, van) were used as controls. Results showed that TRQ could target cell division compared to vancomycin. Scale bar, 100 nm. **(C)** GTPase activity assay of *S. aureus* FtsZ. Both curcumin and TRQ (1/16 dilution) were shown to increase GTPase activity. The experiment was performed three times. Error bar represents the mean ± SD.

The most remarkable finding of our global transcriptional analysis was the reduction of the expression of genes involved in cell division (**Table S1** and **Figure 1C**). It showed that genes encoding cell division proteins FtsZ, FtsL, FtsW and its homologue RodA were all downregulated. As we have mentioned earlier, FtsZ has received extensive attention for the generation of antibacterial agents against pathogens since it is highly conserved across nearly all prokaryotes (Lampson and Kapoor, 2006; Lock and Harry, 2008; Anderson et al., 2012; Ruiz-Avila et al., 2013). Therefore, we envision that TRQ would inhibit the cell division process of *S. aureus* prompting to examine the cellular architecture of *S. aureus* cells after exposure to TRQ. Thus, transmission electron microscopy (TEM) and structured illumination microscopy (SIM) were used to examine *S. aureus* cellular architecture after exposure TRQ. Wheat germ agglutinin conjugate was used to label Z-ring due to its interaction with peptidoglycan, and Nile red was used for membrane labeling. As can be seen from **Figure 2A**, TRQ treatment did not lead to the disruption of the cell membrane but resulted in disappearance of typical Z-ring in the mid-cell of 98.1% of cells (n = 500), probably due to disassembly of FtsZ filaments in the exposed cells. By contrast, the mock cells showed typical mid-cell septum (Z-ring) (80.5%, n = 300) as well as non-typical mid-cell septum (19.5%, n = 300). However, very few disassembly of Z-ring was observed in mock cells. TEM was then used to compare the morphology of *S. aureus* cells after exposure to TRQ. Consistent with SIM results, TRQ treatment caused cell division defects by blocking the assembly of Z-ring compared to that of mock cells and another cell wall-targeting antibiotic, vancomycin (Wang and Sun, 2021), both of which showed typical mid-cell septum (Z-ring) (**Figure 2B**).

To further assess the effect of TRQ on FtsZ filament assembly, we examined its GTPase activity *in vitro* and determined whether TRQ could influence its activity. As can be seen from **Figure 2C**, we found that TRQ treatment led to enhanced activity of GTPase activity of FtsZ compared to the mock group. This could result in the reduced stability of FtsZ filaments and finally lead to the disassembly of Z-ring after TRQ exposure. Our result was in agreement with previous conclusions that enhanced GTPase activity of FtsZ led to failure of Z-ring assembly (Rai et al., 2008; Kumar et al., 2011; Groundwater et al., 2017). Curcumin was used as a positive control (Groundwater et al., 2017), and showed elevated levels of GTPase activity as compared to untreated control (**Figure 2C**).

Altogether, we have demonstrated that TRQ exposure led to limit or inhibit the formation of Z-ring in *S. aureus* to interrupt cell division by elevating GTPase activity.

### TRQ Downregulated Expression of Genes Involved in Virulence of *S. aureus*

Another interesting finding of the transcriptional analysis was the reduction of virulence gene expression. We found that multiple genes involved in virulence factor production showed a drastic decrease at transcriptional level upon TRQ treatment compared to that of mock control **(** **Table S1****)**. Particularly, genes encoding hemolysin (*hla*), autolysin (*atl*), and immunoglobulin-binding protein (*sbi*) were downregulated after TRQ treatment (**Figure 1C**). Therefore, we aimed to determine the biological processes related to these genes by phenotypic analysis.

**Figure 3:**
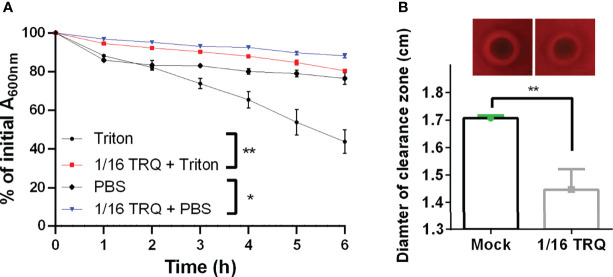
TRQ attenuates autolysis and hemolysis in *S. aureus*. **(A)** Effect of the TRQ (1/16 dilution) on Triton X-100 induced autolysis. The autolysis activity with and without 0.05% Triton X-100 was monitored by measuring OD_600_ over time. PBS control group also showed a similar trend compared to that of Triton X-100 treatment. The experiment was performed three times. Error bar represents the mean ± SD. *P < 0.05; **P < 0.01. **(B)** Hemolysis on sheep blood agar. *S. aureus* strains tested with TRQ (1/16 dilution) were spotted on a 5% (v/v) sheep blood agar plate. Clearance zones indicate hemolysis and were measured. The experiment was performed three times. Error bar represents the mean ± SD. **P < 0.01.

As can be seen from **Figure 3A**, autolysis was determined in two different conditions, one with Triton X-100 induction and the other with PBS buffer, similar to natural condition. We found that TRQ treatment leads to inhibition of autolysis as compared to that of mock under both conditions. Triton X-100 induction gave rise to higher autolysis rate compared to PBS treatment. Furthermore, we have examined the effect of TRQ treatment on the hemolysin activity by using sheep blood agar plate assay. We observed a clear hemolytic halo around bacterial colonies and TRQ-treated cells showed significantly reduced activity of hemolysin, indicating that TRQ could inhibit hemolysin activity *in vitro* (**Figure 3B**). Both phenotypic analyses showed good correlation with RNA-seq analysis and demonstrated that TRQ affected *S. aureus* pathogenesis *in vitro*.

In addition, we have noticed that several genes, including several global virulence regulators such as *mgrA*, *sarZ*, and *agrA*, which are involved in regulation of virulence factors were downregulated following TRQ treatment (**Table 1**). In addition, several TCSs such as SaeSR, KdpDE, and HptSR, were significantly repressed after TRQ treatment. These findings suggest that TRQ attenuated virulence through inhibition of the expression of these global regulators.

**Table 1:** Differential expression of genes encoding transduction systems in *S. aureus*.

Locus tag^a^	Gene^a^	Gene product^a^	Log2FC^b^
SACOL_RS03830	*mgrA*	MarR family transcriptional regulator	-1.99
SACOL_RS07115	*msrR*	regulatory protein MsrR	-1.76
SACOL_RS03930	*saeS* ^c^	two-component sensor histidine kinase	-1.73
SACOL_RS10830	*kdpD* ^c^	sensor histidine kinase	-1.62
SACOL_RS01030	*hptS* ^c^	sensor histidine kinase	-1.59
SACOL_RS12510	*sarZ*	transcriptional regulator	-1.48
SACOL_RS10835	*kdpE* ^c^	DNA-binding response regulator	-1.42
SACOL_RS06765	*glnR*	MerR family transcriptional regulator	-1.37
SACOL_RS11185	*czrA*	transcriptional regulator	-1.32
SACOL_RS01585	*nanR*	MurR/RpiR family transcriptional regulator	-1.19
SACOL_RS10580	*agrA*	DNA-binding response regulator	-1.18
SACOL_RS03935	*saeR* ^c^	DNA-binding response regulator	-1.15
SACOL_RS01025	*hptR* ^c^	DNA-binding response regulator	-1.06
SACOL_RS01245	*lytR*	DNA-binding response regulator	-1.06
SACOL_RS05120	*spxA*	regulatory protein Spx	1.05
SACOL_RS00125	*walK*	cell wall metabolism sensor histidine kinase WalK	1.15
SACOL_RS03755	*ccpE*	LysR family transcriptional regulator	1.26
SACOL_RS03475	*sarA*	transcriptional regulator	1.27
SACOL_RS12225	*hutR*	LysR family transcriptional regulator	1.38
SACOL_RS10030	*perR*	transcriptional repressor	1.44
SACOL_RS07820	*srrB*	two-component sensor histidine kinase	1.49
SACOL_RS01305	*rbsR*	LacI family transcriptional regulator	1.49
SACOL_RS02035	*mepR*	multidrug efflux MATE transporter transcriptional repressor MepR	1.52
SACOL_RS07230	*phoU* ^c^	phosphate transport system regulatory protein PhoU	1.54
SACOL_RS12360	*tcaR*	transcriptional regulator	1.80
SACOL_RS08890	*phoR* ^c^	sensor histidine kinase	2.12
SACOL_RS08355	*hrcA*	HrcA family transcriptional regulator	2.89
SACOL_RS13895	*arcR*	transcriptional regulator	3.88

a Locus tag, gene name, and gene product were extracted from AureoWiki (Fuchs et al., 2018).

b FC, fold change (log2 ratio).

c The expression of these genes have been verified by RT-PCR.

### TRQ Attenuated *S. aureus* Infection *In Vivo*

**Figure 4:**
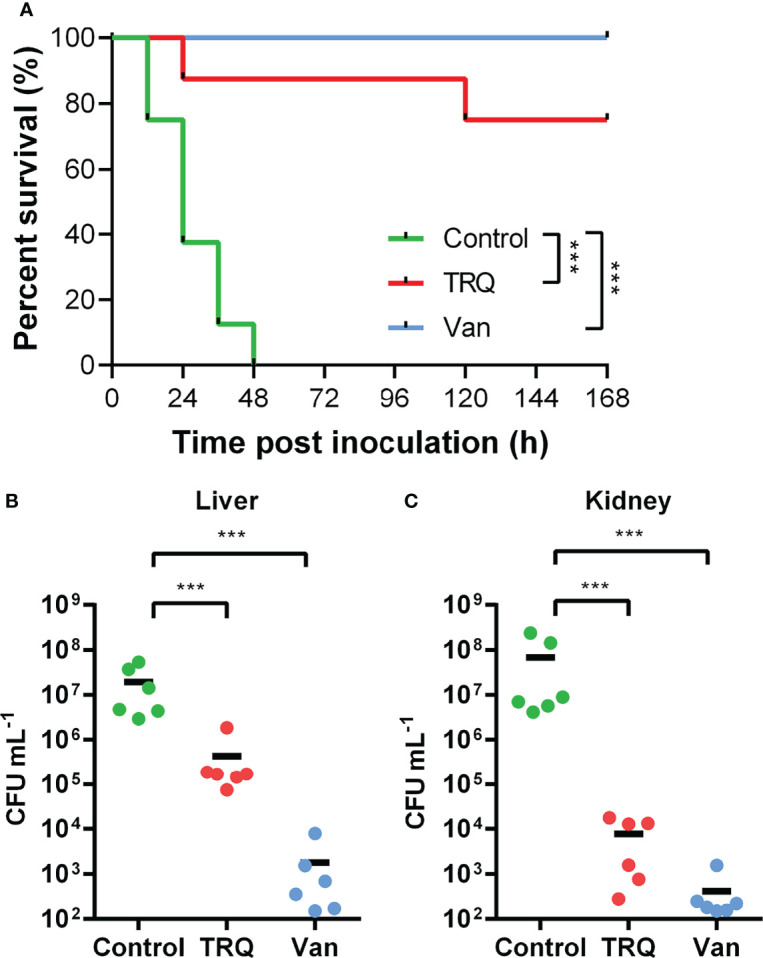
TRQ attenuates *S. aureus* virulence in a murine infection model. The control, TRQ-treated bacteria (TRQ, 1/16 dilution), and vancomycin-treated bacteria (Van) were used to infect 6 mice *via* intravenous injection. **(A)** Effect of TRQ in protecting mice (n=6) from lethal S. aureus infection. ***P < 0.001; log-rank test. After 7 d post-infection, *S. aureus* colonization in murine liver **(B)** or kidney **(C)** was enumerated. Each circle represents one mouse. The horizontal black line represents the mean log_10_ CFU on the *y* axis. The statistical difference between control and TRQ-treated group or control and vancomycin-treated group was determined using Student’s *t*-test. ***P < 0.001.

Previously, we have shown that TRQ formula efficiently protected *Caenorhabditis elegans* from killing by *P. aeruginosa* (Yang et al., 2020). In this study, we have observed several genes showing altered changes in transcription upon TRQ treatment (**Figure 1** and **Table S1** and 1), and we wondered whether these changes may contribute to virulence in an established murine intraperitoneal systemic model of infection (**Figure 4**). Bacteria [untreated control/control, TRQ-treated group/TRQ, and an antibiotics treatment control/Van; 10^6^ colony forming units (CFU)] were injected peritoneally into mice (n = 6) and murine short-term survival analysis were conducted. As shown in **Figure 4A**, we found that *S. aureus* alone was virulent to the animals since more than 50% of the population died after one day treatment and 100% after two days. In contrast, TRQ-treated *S. aureus* cells caused significant increase in animal survival. As a control, vancomycin treatment similarly led to an increase in animal survival comparable to that of TRQ treatment. Bacterial loads in liver (**Figure 4B**) and kidney (**Figure 4C**) were enumerated after establishment of bacterial infection. As expected, TRQ treatment caused a significant reduction of bacterial load in both liver (2 logs) and kidney organs (4 logs). As a positive control, bacterial loads in livers and kidneys were dramatically reduced following vancomycin treatment (4 and 5 logs, respectively). These results indicate that TRQ is a promising anti-virulence agent against *S. aureus* in a murine model.

## Discussion

*S. aureus* is one of human life-threating pathogens that cause significant mortality and morbidity (Knox et al., 2015). The pathogenicity of *S. aureus* is largely dependent on the production of virulence factors such as proteases, hemolysins, and immune-modulatory factors (Kuroda et al., 2001; Foster, 2004; Foster, 2005). The expression of these factors is controlled by regulatory systems including global regulators such as SarZ and MgrA, as well as TCSs, among which SaeRS, SrrAB, WalKR, and LytRS systems (Bronner et al., 2004; Chen et al., 2006; Chen et al., 2009; Delaune et al., 2012). Novel treatments have been developed based on the understandings of these regulatory systems, since there is growing evidences that virulence attenuation can lead to significant inhibition of infections (Gordon et al., 2013). Several compounds including benzobromarone against AgrA and 5,5-methylenedisalicyclic acid against MgrA-DNA interaction, were shown to reduce infections caused by *S. aureus* in animal models (Gordon et al., 2013).

Previously, we have used one of the Chinese traditional medicines named TRQ injection to study its inhibition of biofilm formation of *S. aureus* and found that it suppressed this chronic infection phenotype in a mechanism different from penicillin (Wang et al., 2011). More recently, we have shown that TRQ treatment could efficiently inhibit quorum sensing systems and this attenuation was partially dependent on the suppression of upstream TCSs in *P. aeruginosa* (Yang et al., 2020). To investigate further whether TRQ could have a similar inhibition effect on *S. aureus*-mediated infection, we used transcriptome analysis to uncover the underlying mechanisms of action. Interestingly, TRQ was effectively shown to inhibit the expression of genes encoding virulence factors, transcriptional regulators, and cell division proteins in *S. aureus* at sub-minimum inhibitory concentrations (sub-MIC) *in vitro* and *in vivo* (Wang et al., 2011). Therefore, TRQ could repress the pathogenesis of both Gram-negative and -positive bacteria. Most probably, this would function through distinct QS systems in *S. aureus* and *P. aeruginosa*. However, there are some difference between two studies. For example, when we treated *P. aeruginosa* with TRQ, we found that the most significant change was related to QS systems since a wide array of QS genes were downregulated (Yang et al., 2020). As for TRQ-treated *S. aureus*, we noticed that TRQ targeted not only QS systems but cell division system. Therefore, it would be interesting to search for more common or different targets to further elucidate the mode of action of TRQ.

The most striking finding in our study is the effect of TRQ on cell division. Through combinatory methods, we have shown that several genes involved in this process were downregulated, including FtsZ, FtsL, FtsW, and its homologue RodA (**Figures 1**, **2**), suggesting that the cell division machinery may be affected following TRQ exposure. Using both SIM and TEM analyses, we observed abnormal Z-ring formation and cell division during TRQ treatment compared to control and vancomycin treatment. We further explained this by examining FtsZ GTPase activity and found that TRQ could enhance GTPase activity of FtsZ as compared to curcumin, a compound that has been previously shown to significantly enhance the GTPase activity of FtsZ and destroy the Z-ring formation in *Bacillus subtilis* (Groundwater et al., 2017). We therefore envisioned that similar to curcumin, TRQ might inhibit the assembly of FtsZ most probably *via* increasing the GTP hydrolysis rate and thus enhance the GTPase activity (Groundwater et al., 2017). This finding has extended our understanding of the mode of action of TRQ against bacterial infections. FtsZ serves as an appealing target for the development of antibiotics and plays important roles in bacterial cell division (cytokinesis) (Ruiz-Avila et al., 2013; Sass and Brotz-Oesterhelt, 2013). In the future, it would be necessary to identify the effective components related to FtsZ inhibition.

Another interesting finding from this study is that TRQ could potentially affect the expression of a large repertoire of genes involved in pathogenesis, such as genes encoding hemolysin (*hla*) and autolysin (*atl*). In addition, several two-component systems including SaeSR, KdpDE, and HptSR were also downregulated by TRQ treatment. This finding has pointed out that TRQ could be used as an effective anti-virulence agent, and further murine model analysis has proved this notion. It is coincident that TRQ also targets TCSs to mitigate the expression of quorum sensing systems in *P. aeruginosa*, suggesting that TRQ targets TCSs in both Gram-negative and -positive bacteria to inhibit their virulence. We will further unravel this mechanism and provide insights into the mode of action of TRQ against TCSs in bacteria.

Overall, we have primarily uncovered the mode of action of TRQ against *S. aureus*-associated infections. Our findings lead to a novel notion that TRQ not only targets virulence factors but also affects the cell division, thus leading to cell death.

## Materials and Methods

### Bacterial Strains, Culture Conditions, and Chemicals

Methicillin-resistant S*. aureus* ATCC 43300 (MRSA) strain was grown in Lysogeny broth (LB) or tryptone soya both (TSB) with aeration at 37°C. When required, LB agar plates were used to streak bacterial colonies. TRQ injection is a second-class new Traditional Chinese Medicine (Approval No. Z20030054, Shanghai Kaibao Pharmaceutical Company, China).

### Growth Curve

Overnight cultures of bacterial strains in LB were diluted (1:100) in 3 mL LB medium and precultures were incubated aerobically at 37^о^C in a shaker at 200 rpm to an OD_600_ of 0.5. The precultures were further diluted (1:100) in 1 mL LB medium. Growth was then analysed in 10x10-well microtitre plates containing 294 µL LB medium to which 6 µL of diluted precultures containing 10^5^ cells were added to obtain a final 1:5000 dilution. Control wells contained only the growth medium without bacteria. TRQ treatment was prepared in a 1:16, 1:32, 1:64 dilutions using LB medium. The microtitre plates were incubated for 72 h at 37°C in a Bioscreen incubator (Life Technologies, Finland) using the following settings: shaking for 20 s every 3 min and absorbance measured every 30 min at 600 nm. Each culture was prepared in triplicate.

### RNA Extraction

Overnight cultures of *S. aureus* in LB were used to inoculate fresh LB medium in a 1:1000 dilution in the absence and presence of TRQ (1:16 dilution ratio). After 12 h of incubation, one mL of culture was fixed immediately with 2 mL of RNA Protect Reagent (Qiagen), following the manufacturer’s instructions, and the fixed cell pellets were frozen at -80°C until further use. All experiments were performed in triplicate. Total RNA was extracted using TRIzol^®^ Reagent according to the manufacturer’s instructions (Invitrogen) and genomic DNA was removed using RNase-free DNase I (TaKaRa). Then RNA quality was determined using 2100 Bioanalyzer (Agilent) and quantified using the ND-2000 (NanoDrop Technologies). High-quality RNA sample (OD_260_/OD_280 =_ 1.8~2.2, OD_260_/OD_230_≥2.0, RIN≥6.5, 28S:18S≥1.0, >10 μg) was used for qRT-PCR.

### Library Preparation, and Illumina HiSeq Sequencing

RNA-seq strand-specific libraries were prepared following TruSeq RNA sample preparation Kit from Illumina (San Diego, CA), using 5 μg of total RNA. Briefly, rRNA was removed by RiboZero rRNA removal kit (Epicenter), fragmented using fragmentation buffer. cDNA synthesis, end repair, A-base addition, and ligation of the Illumina-indexed adaptors were performed according to Illumina’s protocol. Libraries were then size selected for cDNA target fragments of 200~300 bp on 2% Low Range Ultra Agarose followed by PCR amplified using Phusion DNA polymerase (NEB) for 15 PCR cycles. After quantification by TBS380 Mini-Fluorometer, paired-end libraries were sequenced by BGI Biotechnology Co., Ltd (Shenzhen, China) with the BGISEQ-500 PE 2 × 50 bp read length.

### Reads Quality Control and Mapping

The raw paired end reads were trimmed and quality controlled by Trimmomatic with default parameters (Bolger et al., 2014). Then clean reads were separately aligned to the reference genome (*S. aureus* COL, Accession number NC_002516) with orientation mode using Rockhopper software (Mcclure et al., 2013; Tjaden, 2015), which was a comprehensive and user-friendly system for computational analysis of bacterial RNA-seq data. As an input, Rockhopper takes RNA sequencing reads generated by high-throughput sequencing technology to calculate gene expression levels with default parameters.

### Differential Expression Analysis and Functional Enrichment

To identify DEGs (differential expression genes) between two different samples, the expression level for each transcript was calculated using the fragments per kilobase of reads per million mapped reads (RPKM) method. The method edgeR was used for differential expression analysis (Robinson et al., 2010). The DEGs between two samples were selected using the following criteria: the logarithmic of fold change was greater than 2 and the false discovery rate (FDR) should be less than 0.05. To understand the functions of those differential expressed genes, GO functional enrichment and KEGG pathway analysis were carried out by Goatools (Klopfenstein et al., 2018) and KOBAS (Xie et al., 2011), respectively. DEGs were significantly enriched in GO terms and metabolic pathways when their Bonferroni-corrected *P*-value was less than 0.05. The RNA-seq datasets have been deposited in National Center for Biotechnology Information (NCBI) with an accession number GSE162107.

### qRT-PCR

Bacterial cells were harvested in similar condition to RNA-seq analysis and collected in RNA protect bacteria reagent (Qiagen). Total RNA was isolated and residual DNA was removed by treatment with DNase I (Takara). The purity and concentration of the RNA was determined by gel electrophoresis and spectrophotometry. Reverse transcription was performed using 1 µg of total RNA and the first-strand cDNA synthesis kit from GE Healthcare as indicated by the manufacturer. Quantitative real-time PCR was carried out with 50 ng of first-strand cDNA in a total volume of 25 µL to assess the effect of TRQ on expression of the selected genes (**Table S2**). Optimal primer concentrations were determined and standard curves for each PCR reaction were performed prior to relative quantification analysis. qRT-PCR was performed in a Bio-Rad (Hercules, CA, USA) iCycler with Bio-Rad iQ SYBR Green Supermix. For all primer sets, the following cycling parameters were used: 94°C for 3 min followed by 40 cycles of 94°C for 30 s, 55°C for 45 s and 72°C for 30 s, followed by 72°C for 7 min. Fold changes were determined using the comparative threshold cycle method with the housekeeping gene 16S rRNA (Schmittgen and Livak, 2008). All experiments were carried out in triplicate.

### Transmission Electron Microscopy (TEM)

*S. aureus* was grown in LB media containing TRQ (final concentration: 1/8), or vancomycin (final concentration: 2 µg/mL), and an untreated control was included. The cultures were treated for 2.5 h at 37°C with shaking, after which the bacteria were collected by centrifugation and washed twice with PBS. Pellets were fixed overnight at room temperature in 2.5% (*v*/*v*) glutaraldehyde and post-fixed with 1% OsO4 solution for 60 min. The samples were dehydrated using increasing concentrations of ethanol (50, 70,95, and 100%) and then embedded in epon TAAB-812. The samples were cut into ultrathin sections using an ultra-microtome and collected on a nickel grid. The sections were stained using 3% uranyl acetate for 14 min at 60°C followed by a wash using water and then stained using lead citrate for 6 min at room temperature. Finally, the samples were washed in 20 mM NaOH and water and then dried. The sections were viewed under transmission electron microscope (TEM) (H-7500, Hitachi, Japan). The experiment was carried out using two biological replicates.

### Structured Illumination Microscopy (SIM)

*S. aureus* cultures grown at 37°C in LB medium were diluted 1:1000 into fresh LB medium and grown until mid-logarithmic phase for 5 h at 37°C. Subsequently, cells were grown in LB media containing 1/8 TRQ or 2 µg/mLVan for 30 min at 37°C. Then, cells were stained with 2 mg/L wheat germ agglutinin Alexa Fluor 488 conjugate (WGA-488,Invitrogen) at 37°C with agitation for 10 min. Unbound dye was removed from the media by washing the cells with PBS and cells were then incubated with Nile Red (10 mg/L) for 10 min at room temperature and placed on an agarose pad containing 50% LB in PBS. For structured illumination microscopy, cells were viewed using a DeltaVision OMX (Applied Precision/GE Healthcare) comprising an OMX optical microscope (version 3), using a 561 nm laser for Nile Red, 488 nm laser for WGA-488, and 100 ms exposure.

### GTPase Assay

The effect of TRQ on the GTPase activity of FtsZ was determined using malachite green ammonium molybdate as described previously (Groundwater et al., 2017). Briefly, FtsZ (6 μM) was incubated without and with 1/16 TRQ in buffer A (25 mM PIPES, 5 mM MgCl2, 50 mM KCl, pH 7.2) on ice for 15 min. Polymerization was triggered by adding 1 mM GTP to the reaction mixtures that were further incubated at 37°C for 10 min. The reaction was quenched by using 7 M perchloric acid (10%, v/v) and then 40 μL of the reaction mixture were incubated with 900 μL of freshly prepared malachite green ammonium molybdate solution (0.045% malachite green, 4.2% ammonium molybdate, and 0.02% Triton X-100) at room temperature for 30 min. The molar number of inorganic phosphate released was calculated by measuring the absorbance at 650 nm and quantified from a standard phosphate curve. The experiment was performed independently for three times.

### Autolysis Assay

Triton X-100-induced autolysis assays were performed as previously described with modifications (Manna et al., 2004; Chen et al., 2009). Briefly, overnight cultures were diluted 1:100 with 5 mL of TSB containing 1 M NaCl and grown at 37°C with shaking until OD_600nm_ reached 1.0. Cells were pelleted by centrifugation, washed twice with ice-cold distilled water, and then resuspended in 50 mM phosphate buffered saline (PBS, pH 7.2) supplemented with and without 0.05% Triton X-100 (v/v). Bacterial cells were then incubated at 37°C with shaking and the autolysis activity was determined by measuring OD600 over time. All assays were conducted in triplicate.

### Hemolysin Analysis

The TRQ-treated and mock bacterial strains were spotted and inoculated on a 5% (v/v) sheep blood agar plate at 37°C for 24 h. Clearance of zones indicates hemolysis. All assays were performed in triplicate.

### Animal Usage Declaration

6~8 week old male BALB/c mice were purchased from Beijing Vital River Laboratory Animal Technology Co. Ltd (No. CNAS LA0004). Mice were adapted to standardized environmental conditions (Temperature=23 ± 2°C; humidity=55 ± 10%) for one week prior to infection. Mice were maintained in strict accordance with the regulations for the Administration of Affairs Concerning Experimental Animals approved by the State Council of People’s Republic of China (GB/T 35892-2018). The animal study protocols were performed in accordance with the relevant guidelines and regulations (SYXK(Beijing)2021-0017, Experimental Research Center, China Academy of Chinese Medical Sciences). The laboratory animal usage license number is SCXK(Beijing)2016-0006 and certified by Beijing Vital River Laboratory Animal Technology Co. Ltd.

### Animal Infection Assay

*S. aureus* were grown at 37°C overnight in LB medium. The cultures were diluted 1:100 with fresh LB and then incubated at 37°C for 2 h until OD_600_ reached 1.0. Bacteria were collected by centrifugation, washed, and resuspended in PBS to an OD_600_ of 0.4. Viable staphylococci were enumerated by colony formation on LB plates to measure the infection dose (10^7^ CFU). Male BALB/c mice (6~8 weeks, 6 per group) were infected with a dose of 10^7^ CFU bacterial suspension *via* peritoneal injection. After 1 h post infection, mice were treated with a single dose of Van (10 mg kg^-1^), TRQ (5 mL kg^-1^) alone *via* tail intravenous injection. Mice were killed by CO_2_ asphyxiation 7 d after injection, and kidneys and livers were removed. The organs were homogenized in 1 mL of PBS, and 10 mL of dilution of the homogenates was plated on LB plates.

### Statistical Analysis

The data of qRT-PCR, virulence factor production, and virulence tests were analysed by one-way ANOVA. Student’s *t*-test was used when one-way ANOVA revealed significant differences (*P* < 0.05). Survival data were analysed *via* the Kaplan-Meier method and the log-rank test was used to compare the significant differences between subgroups (*P* < 0.01). All statistical analyses were performed with GraphPad Prism statistical software (GraphPad Software, La Jolla, USA) with the assistance of Excel (Microsoft).

## Data Availability Statement

The datasets presented in this study can be found in online repositories. The names of the repository/repositories and accession number(s) can be found below: https://www.ncbi.nlm.nih.gov/geo/, GSE162107.

## Ethics Statement

The animal study was reviewed and approved by Experimental Research Center, China Academy of Chinese Medical Sciences.

## Author Contributions

YW conceived the project and supervised the study. WY, KC, QT, SM, YS, and QW performed the experiments and analyzed the data. GH, DL, and LL performed experiments, provided the reagents, technical assistance and interpretation of data for the project. BB, SC, and YHW provided technical assistance and interpretation of data for the project. WY, QW, SC, and YW wrote the draft. All authors added comments and corrections and approved the final version to be published.

## Funding

This work was supported by National Mega-project for Innovative Drugs (2019ZX09721001-005-002), Office of International Cooperation, China Academy of Chinese Medical Sciences (GH2018001), Academician Expert Workstation, National Key Research and Development Program of China (No. 2019YFC1709305), and Kaibao Pharmaceutical Company, Shanghai, and Basic Scientific Research Fund from Ministry of Finance of China (No. ZZ2017001).

## Conflict of Interest

The authors declare that the research was conducted in the absence of any commercial or financial relationships that could be construed as a potential conflict of interest.

## Publisher’s Note

All claims expressed in this article are solely those of the authors and do not necessarily represent those of their affiliated organizations, or those of the publisher, the editors and the reviewers. Any product that may be evaluated in this article, or claim that may be made by its manufacturer, is not guaranteed or endorsed by the publisher.

## Supplementary Material

Supplementary Table 1 TRQ-treated *S. aureus* regulon in exponential growth phase. **(A)** Locus tag, gene name, gene product, and functional classification were extracted from AureoWiki (Fuchs et al., 2018). **(B)** FC, fold change (log2 ratio).

Supplementary Table 2 RT-PCR primers used in this study.
